# Current Research on Infectious Diseases of Domestic Animals from a One Health Perspective

**DOI:** 10.3390/pathogens12050724

**Published:** 2023-05-17

**Authors:** Alfonso Zecconi

**Affiliations:** One Health Unit, Department of Biomedical, Surgical and Dental Sciences, Università degli Studi di Milano, 20133 Milan, Italy; alfonso.zecconi@unimi.it

One Health is a well-known strategy for promoting and developing interdisciplinary collaboration across all aspects of health in human, animal, and environmental domains. This approach is important when facing challenges represented by zoonotic pathogens or related to the spread of antimicrobial resistance (AMR). This Special Issue of *Pathogens* aimed to collect original research and review articles on all aspects of transmissible diseases of animals and on AMR to increase our knowledge on the interactions between animal pathogens and the global health issues from a One Health perspective.

By chance, the Special Issue was opened at the beginning of the COVID-19 pandemic, and it was closed at the end. Therefore, studies focusing on this topic from an animal perspective were included. The first paper by Pozzi et al. [1] showed that the experiences amassed during the long-lasting war against Coronaviruses in animal medicine provide some useful information that may be applicable to COVID-19, such as the control of air inlets in healthcare facilities. Another paper by Soltane et al. [2] investigated the potential application of structural analogues prepared from natural maslinic and oleanolic acids against the SARS-CoV-2 main protease as a tool for developing new therapeutical approaches, with interesting results.

Among the zoonotic respiratory diseases, avian flu represents one of the most important. Despite our vast knowledge on the epidemiology of this virus in avian species, relatively less is known about cases when other species specific to certain geographical areas are considered. The potential role of dromedaries in the spread on Influenza A virus was investigated in a paper by Adamu et al. [3], exploring the seroprevalence of this virus. The importance of these viruses for a One Health approach is furthermore supported by the results of the study by Li et al. [4], showing that clade 2.3.4.4 H5N6 HPAIVs exhibited a high replication efficiency in both avian and mammal cells, with high pathogenicity in both mice and chickens, thus demonstrating its high risk to public health.

For the last three years, the pandemic has shadowed other important problems affecting global health originating from AMR. This topic was covered in a paper by Hamame et al. [5] on the role of pet animals in the spread of colistin resistance to humans due to the presence of *mcr* genes in pets. The results confirmed the risk of transmission of the resistance gene from animals to humans. Other bacteria that are common to animals and humans are extra-intestinal pathogenic *E. coli* (ExPEC). Their resistance patterns and role in the spread of AMR were considered in another review by Sora et al. [6]. 

Wild animals represent a known problem in the spread of zoonotic disease. This Special Issue includes a paper showing the roles of deer keds, as well as the ectoparasites of these animals, in the transmission of several pathogens, suggesting that keds may be used as biological markers for investigating the prevalence of vector-borne diseases [7]. The cervids are also involved in the spread of the zoonotic enteric protozoan *Balantioides coli*, as reported in the study of Mega et al. [8].

The risk of close contact between humans and wild animals is relatively low, but when pet animals are considered, this risk is much higher. The paper of Anastàcio et al. [9] showed that the monitoring of *C. burnetii* infections in companion animals is an important tool that may be used to prevent human outbreaks, considering the zoonotic potential of owners and veterinarians in contact with infected animals. The importance of proper surveillance was also highlighted in the paper of Pappa et al. [10] on leishmaniasis, which is a neglected tropical disease affecting humans and domesticated animals, with high mortality in endemic countries. 

If data on the potential roles of some animal species in the spread of zoonotic diseases are relatively abundant, there are other topics that need a more detailed investigations, such as the role of free-range cats and dogs as potential carriers of several zoonotic pathogens, as reported by Mesquita et al. [11], or the role of tick-infested sheep in the epidemiology of Borrelia infection, as reported by Athanasiou et al. [12]. Animals and vectors are also involved in the epidemiology of *Bluetongue* virus, and the role of the interface between wild and domesticated animals in spreading this virus was described by Rivera et al. [13].

Early and accurate diagnosis is crucial for the effective surveillance and control of diseases, particularly when zoonotic ones are considered. The results of Tilocca et al. [14] on Paratuberculosis support this statement, suggesting that the evaluation of the immunogenic properties of 10 *Mycobacterium avium subsp. paratuberculosis* proteins could provide a tool for improving the current diagnostic technologies. 

This Special Issue collected many papers on different topics, confirming the need for a transdisciplinary approach to the surveillance and control of diseases, which is one of the main features of One Health.
